# Satellite Telemetry and Long-Range Bat Movements

**DOI:** 10.1371/journal.pone.0014696

**Published:** 2011-02-16

**Authors:** Craig S. Smith, Jonathan H. Epstein, Andrew C. Breed, Raina K. Plowright, Kevin J. Olival, Carol de Jong, Peter Daszak, Hume E. Field

**Affiliations:** 1 Biosecurity Sciences Laboratory, Department of Employment, Biosecurity Queensland, Economic Development & Innovation, Coopers Plains, Queensland, Australia; 2 EcoHealth Alliance, New York City, New York, United States of America; 3 Centre for Epidemiology and Risk Analysis, Veterinary Laboratories Agency, Addlestone, Surrey, United Kingdom; 4 Department of Medicine and Epidemiology, School of Veterinary Medicine, University of California Davis, Davis, California, United States of America; University of Georgia, United States of America

## Abstract

**Background:**

Understanding the long-distance movement of bats has direct relevance to studies of population dynamics, ecology, disease emergence, and conservation.

**Methodology/Principal Findings:**

We developed and trialed several collar and platform terminal transmitter (PTT) combinations on both free-living and captive fruit bats (Family *Pteropodidae*: Genus *Pteropus*). We examined transmitter weight, size, profile and comfort as key determinants of maximized transmitter activity. We then tested the importance of bat-related variables (species size/weight, roosting habitat and behavior) and environmental variables (day-length, rainfall pattern) in determining optimal collar/PTT configuration. We compared battery- and solar-powered PTT performance in various field situations, and found the latter more successful in maintaining voltage on species that roosted higher in the tree canopy, and at lower density, than those that roost more densely and lower in trees. Finally, we trialed transmitter accuracy, and found that actual distance errors and Argos location class error estimates were in broad agreement.

**Conclusions/Significance:**

We conclude that no single collar or transmitter design is optimal for all bat species, and that species size/weight, species ecology and study objectives are key design considerations. Our study provides a strategy for collar and platform choice that will be applicable to a larger number of bat species as transmitter size and weight continue to decrease in the future.

## Introduction

Old-World fruit bats (Family *Pteropodidae*) play a vital ecological role as pollinators and seed dispersers in forest ecosystems [1]. Many species are nomadic, shifting roosting and foraging locations in accordance with food availability [2], [3], [4]. The pattern and magnitude of these nomadic movements are, for the most part, poorly understood [5], but individual movements of over 1,500 kilometers have been reported [6].

Population declines and altered population dynamics have been reported in many fruit bat species, and are likely a response to deforestation and other anthropogenic habitat changes [7]. In some cases, these changes increase the likelihood of contact between fruit bats, domestic animals and humans [8], [9] and are hypothesized to have promoted the emergence of several highly pathogenic zoonotic agents from fruit bats, including Nipah virus in Asia [10], [11], [12], [13] and Hendra virus in Australia [14], [15], [16]. Understanding the movement dynamics of fruit bats informs both their conservation management and the mitigation of disease emergence.

Satellite telemetry has been used to elaborate the frequency and magnitude of fruit bat movements [17], [18], [19]. However, the effective deployment of satellite transmitters on bats poses unique challenges because of their size (typically <1 kg), anatomy (wing membrane stretching from forelimb to hindlimb), roosting behavior (inverted and colonial), and life history traits (mothers carry pups in flight). Further, the weight and dimensions of available platform terminal transmitters (PTTs), notwithstanding recent advances, make ready and effective attachment to bats difficult. These issues threaten both the quality and duration of transmissions, and potentially the health and welfare of tagged individuals.

In this paper we provide the most comprehensive overview of satellite telemetry methods in bats to date, and describe our development and trialing of collar and PTT configurations on both free-living and captive fruit bats (genus *Pteropus*, family *Pteropodidae*), commonly known as flying foxes. We discuss the strengths and weaknesses of each configuration to identify key design determinants affecting satellite telemetry study outcomes, based on successful field trials in Southeast Asia and Australasia.

## Methods

### Study 1: Collar design

**Figure 1:**
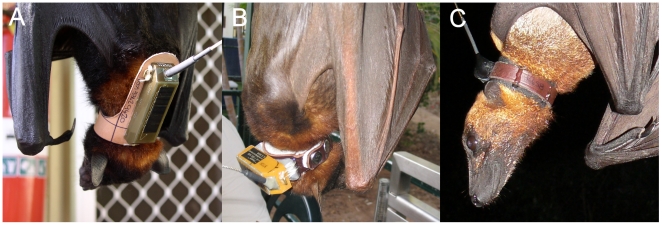
Examples of the three collar designs. A) Collar design 1, an 18 g solar powered PTT deployed on a black flying fox (*Pteropus alecto*) along the dorsal aspect of the neck, between the scapulae and parallel to the axis of the spine. B) Collar design 2, a 12 g solar powered PTT deployed on a little red flying fox (*Pteropus scapulatus*) on the dorsal aspect of the neck and perpendicular to the axis of the spine. C) Collar design 3, a 22 g battery powered PTT deployed on a Bismarck or great flying fox (*Pteropus neohibernicus*) on the dorsal aspect of the neck and perpendicular to the axis of the spine.

We sequentially developed three collar designs using results from field and captive trials to inform subsequent modifications, and incorporating Microwave Telemetry^TM^ PTTs. Design 1 (Fig. 1a) was a modification of a collar and bib design described by Tidemann and Nelson [17] to accommodate the elongated shape of the 18 g solar-powered PTT (62 mm ×18 mm ×12.5 mm) and the 20 g battery-powered PTT (54 mm×18 mm ×17 mm) [20]. It positioned the PTT along the dorsal aspect of the neck, between the scapulae and parallel to the axis of the spine. The one-piece collar and bib was made from 1.4 mm ‘veggie-tan’ leather; the collar was 11 mm wide and the bib was 28 mm wide and long enough to accommodate the PTT. (‘Veggie-tan’ refers to leather treated with tannins of plant origin, and ‘chrome’ (below) to leather treated with chromium sulfate; the latter generally produces softer and more flexible leather). The PTT was attached to the bib with an all-weather contact adhesive (Selleys, Australia) and nylon thread (using the PTT attachment loops). The thread and knots were sealed using a two-part epoxy resin [21]. This design was used with three 18 g solar-powered PTTs and four 20 g battery-powered PTTs (Table 1).

**Table 1:** Study 1 – Transmitter and collar characteristics listed by release date.

Bat/ PTT	Collar Design	PTT Model	Duty Cycle (ON/OFF h)	Species	Release Date	Days Active
A	1	18 g Solar	12/155	*P. alecto*	9/6/2003	121
B	1	18 g Solar	12/155	*P. alecto*	11/10/2003	231
C	1	18 g Solar	12/155	*P. alecto*	17/10/2003	225
D	1	20 g Battery	12/222	*P. vampyrus*	17/12/2003	105
E	1	20 g Battery	12/222	*P. vampyrus*	28/6/2004	2
F	1	20 g Battery	12/222	*P. vampyrus*	8/7/2004	103
G	1	20 g Battery	12/222	*P. vampyrus*	9/7/2004	127
H	2	12 g Solar	7/155	*P. vampyrus*	13/8/2005	42^a^
I	2	20 g Battery	7/107	*P. vampyrus*	11/8/2005	146
J	2	20 g Battery	7/107	*P. vampyrus*	28/10/2005	131
K	2	12 g Solar	7/155	*P. scapulatus*	26/10/2005	57
L	2	12 g Solar	7/155	*P. scapulatus*	26/10/2005	41
M	2	12 g Solar	7/155	*P. scapulatus*	31/10/2005	68
N	2	12 g Solar	7/155	*P. scapulatus*	30/10/2005	27
O	2	20 g Battery	7/155	*P. vampyrus*	12/12/2005	47
P	2	20 g Battery	7/155	*P. vampyrus*	12/12/2005	47
Q	2	12 g Solar	7/155	*P. alecto*	18/7/2006	341
R	3	22 g Battery^d^	8/24^b^ 8/120^c^	*P. neohibernicus*	21/7/2006	62
S	3	22 g Batteryd,e	8/24^b^ 8/120^c^	*P. neohibernicus*	17/7/2006	108
T	2	12 g Solar	7/155	*P. alecto*	29/08/2007	47

a Bat H killed by hunter.

b First 5 duty cycles.

c Duty cycles until end of battery life.

d Implantable PTT used on collar.

e Bat S was an adult female; all other bats were adult males.

Design 2 (Fig. 1b) used a softer ‘chrome’ leather collar, and was initially designed to accommodate a 12 g solar-powered PTT (43 mm ×18 mm ×14 mm) modified by the manufacturer at our request; the antenna was rotated 90° to be perpendicular to the length of the PTT. The collar design positioned the PTT transversely on the dorsal aspect of the neck, perpendicular to the spine. The collar was 1.4 mm thick and 11 mm wide, with a wider section to accommodate the PTT. The PTT was first mounted on a curved bracket of 1 mm thick aluminum (39 mm ×12 mm; curve diameter 32 mm) using a two-part epoxy resin. The bracket was then glued and stitched to the collar as described previously. Sheepskin, with the wool trimmed to 3 mm, was glued to the inside surface of the collar with all-weather contact adhesive. This design was used with six 12 g solar powered PTTs and later, with four (similarly modified) 20 g battery-powered PTTs (Table 1).

Design 3 (Fig. 1c) used a modified proprietary leather cat collar to accommodate a 22 g battery-powered PTT (42 mm ×18 mm ×14 mm) with aerial alignment modified as above. This design positioned the PTT transversely on the dorsal aspect of the neck, perpendicular to the spine, but (because of its compact dimensions) without the need for the bracket used in design 2. The PTT was attached to the 11 mm wide collar using 30 mm heat-shrink tubing, and further secured with nylon thread again embedded in a two-part epoxy resin. The inside of the collar was lined with neoprene that extended 3 mm either side, and was glued and stitched to the collar as described previously. This design was used to deploy two 22 g battery-powered, ‘implantable’ PTTs (Table 1).

All bats were anesthetized prior to collar attachment for animal welfare reasons (to avoid extended physical restraint) and to ensure collar fit was optimized. In Australia, (Bats A–C and K–N), the inhalation agent Isoflurane^TM^ was used as described by Jonsson [22]. In Malaysia (Bats D–J), Timor Leste (Bats O and P) and Papua New Guinea (Bats Q–T), flying foxes were anesthetized using a combination of ketamine hydrochlorine and xylazine injected into the pectoral muscles [23]. Collars were secured with two brass rivets, leaving a 7–8 mm gap between collar and neck to facilitate normal respiration, feeding and grooming, while endeavoring to ensure that the collar could not slip over the head. Bats were released at their point of capture within 30 minutes of recovery from anesthesia, and within 2 hrs of capture.

### Study 2: Solar-powered PTT assessment

Half of the 20 PTTs deployed were solar-powered (Table 1). Transmission and battery characteristics of these PTTs were compared to those of the 10 battery-powered PTTs. Prior to deployment, we assessed battery-charging efficiency under different light conditions on two 12 g solar-powered PTTs mounted on top of a 4 m wooden mast erected in an open area (in Brisbane, Australia) during August and September 2005. The solar panels of one had a southern aspect and thus were not exposed to direct sunlight; the solar panels of the other faced north, and were exposed to direct sunlight. The intended deployment duty cycle of 7 hrs ON and 155 hrs OFF was used, and voltage recorded at the beginning of each duty cycle over a 4-week period. Voltage was recorded from the first signal received which contained sensor data, and within the first 2 hrs of the ON duty cycle. Subsequently, after fully recharging by exposure to a 100 Watt incandescent light bulb at a distance of 15 cm for 12 hours, these PTTs were deployed on two male *P. scapulatus* (Bats K and L) in October 2005. Data were collected from two other 12 g solar-powered PTTs deployed on additional species to assess whether roosting density and roosting height influenced solar-powered PTT performance. One was deployed on Bat H, a male *P. vampyrus* in southern Peninsular Malaysia; the other on Bat Q, a male *P. alecto* from southern Papua New Guinea.

### Study 3: Location Class Error

PTTs transmit signals to Argos receivers on National Oceanic and Atmospheric Administration polar-orbiting environmental satellites which provide full global coverage [24]. These satellites, orbiting at an altitude of 850 km, re-transmit the signals to Argos centers (in France, USA, Australia, Japan and Peru) where they are processed and the location of the PTT is calculated. The locations are assigned an error or LC, defined by Argos as 3 (<150 m), 2 (150<300 m), 1 (300<1000 m), 0 (>1000 m), and A, B and Z (no error range calculable). Locations are typically calculated by the Argos processing centers after receiving a minimum of 4 signals from a PTT, with an interval of at least 240 seconds [25]. An important source of error originates from the stability of the PTT oscillator, which is largely influenced by temperature. Argos certification requires a stability of 4 Hz over 20 min, which would result in 65% of errors being <1100 m [25].

The accuracy of the Argos location class (LC) error was assessed by comparing the difference between 49 Argos reported locations from 6 PTTs over 4 duty cycles with their actual (known) location (atop the 4 m wooden mast described in Study 2), determined using an eMap GPS [26]. Location data were plotted using Arcview 3.3 [27] using Argos Tools [28] and the actual error measured.

### Study 4: Captive bat studies

Two separate trials on captive bats were undertaken to assess any effect of collaring on bat behavior and health. In the first trial, prior to deployment of the first PTT, five wild-caught *P. alecto* (recruited for an unrelated study) were fitted with Design 2 collars mounted with mock 12 g solar powered PTT, and monitored for up to 28 days. Bats 1, 3 and 4 had collars made from ‘chrome’ leather, lined with sheepskin trimmed to 10 mm. Bat 2′s collar was unlined ‘chrome’ leather, and Bat 5′s collar was unlined ‘veggie-tan’ leather. The bats were observed daily, and the skin underneath and around the collar was closely examined under inhalation anesthesia (as described above) on days 1–5, 7, 9, 14, 21 and 28. On Day 9, all collars were rotated 180° so that the PTT was on the ventral surface of the neck, to establish if bats could dorsally re-position the PTT. The bats were observed after recovery and on subsequent days.

In the second trial, ten rescued and recuperating wild flying foxes (8 *P. scapulatus* and 2 *P. conspicillatus*) were housed communally in a large flight enclosure at a wildlife rehabilitation facility. These bats were fitted with a real or dummy transmitter on a Design 2 collar and observed for up to 28 weeks.

### Statistical Analysis

Location data was received from the Argos processing center using Telnet Client and Telnet Inferno [29] or received by email from the Argos Automatic Distribution Service. In Studies 1 and 2, range and median values of transmitter activity were calculated. In Study 2, a two-sample *t*-test was used to compare battery voltage under different light intensities. In Study 3, the mean error and standard variation between known actual PTT locations and Argos reported locations were calculated. Statistical analysis was performed using the Data Analysis package in Microsoft Office EXCEL 2003 [30] or GenStat 9^th^ Version [31].

### Permits and Animal Welfare

All studies performed on live animals followed American Society of Mammalogists guidelines [32] and were approved by the Queensland Department of Industries and Fisheries and The University of Queensland animal ethics committees, the Queensland Parks and Wildlife Service, and respective wildlife agencies in Malaysia, Papua New Guinea and Timor-Leste.

## Results

### Study 1: Collar Design

Observations prior to release showed all three collar designs to be well-tolerated by bats; no scratching, biting or panic behavior was observed in any bat. All collar designs maintained the PTT in the desired dorsal position. With Design 1, there was a tendency for the bib to hinge outwards from the collar under the weight of the PPT when the bat was roosting (upside-down). With Design 2, the mounting bracket allowed the collar to conform well to the curvature of the neck of the bat, but moved the centre of gravity of the PTT 10–12 mm out from the neck, leading to a tendency (at roost) for the collar to flex in the transverse plane with the weight of the PTT. This tendency was not evident with Design 3.

The range of transmitter activity on Designs 1, 2, and 3 was 2–231 days (median 121 days), 27–341 days (median 47), and 62–108 days (median 85) respectively (Table 1). The range of transmitter activity on 12 g solar, 18 g solar, 20 g battery, and 22 g battery PTTs was 27–341 days (median 47), 121–231 days (median 225), 2–146 days (median 104), and 62–108 days (median 85) respectively (Table 1). The range of transmitter activity on *P. alecto, P. scapulatus, P. neohibernicus,* and *P. vampyrus* was 47–341 days (median 225), 27–68 days (median 34), 62–108 days (median 85), and 2–146 days (median 103) respectively (Table 1).

### Study 2: Solar-powered PTT assessment

The range of transmitter activity on the deployed solar- and battery-powered PTTs was 27–341 days (median 63) and 2–146 days (median 104), respectively. In the pre-deployment assessment, there was a statistically significant difference between the mean weekly battery voltage of the PTT exposed to direct sunlight and the PTT not exposed to direct sunlight (*t* = −32.08, *df*  = 6, *P* = <0.001). The former had a mean of 4.2 volts (*SD*  = 0.012); the latter had a mean of 3.96 volts (*SD*  = 0.01). Mean day-length over the 4-week period was 11 hrs 40 mins.

**Figure 2:**
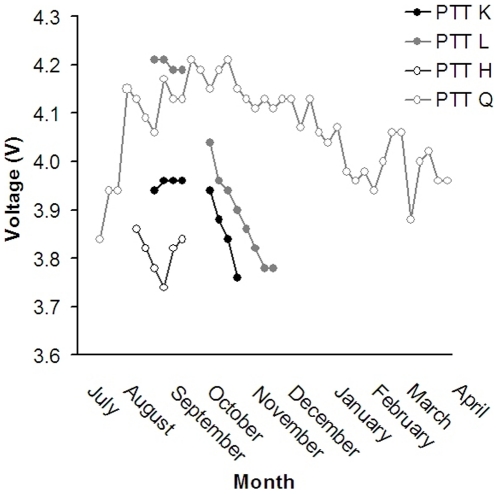
Initial ON cycle voltage (7 h ON/105 h OFF) of four 12 g solar powered PTTs. The trial of PTT K (receiving no direct sunlight) and PTT L (receiving direct sunlight) in August and September 2004 in Brisbane, Australia, and their subsequent deployment on little red flying foxes (*Pteropus scapulatus*) in October 2005 in northern Australia, until December 2005 when their transmission ceased due to low battery voltage. PTT H deployed on a Malayan flying fox (*Pteropus vampyrus*) in August 2005 in southern Peninsular Malaysia before being hunted. PTT Q deployed on a black flying fox (*Pteropus alecto*) in July 2006 in western Papua New Guinea remained active until June 2007.

After deployment, Bats K and L transmitted for 4 and 8 weeks respectively until battery voltage was 3.76 and 3.78 respectively (Fig. 2). Mean day-length in northern Australia over the trial period was 12 hrs 35 mins. Bat H transmitted for 8 weeks, with a mean weekly battery voltage of 3.83 (*SD*  = 0.05). Mean day-length in Peninsular Malaysia over the trial period was 12 hrs 10 mins. Bat H was killed by a hunter (and the PTT returned to the authors) (Fig. 2). Bat Q's PTT maintained a mean weekly voltage of 4.07 V (*SD*  = 0.08, n = 39) during its 48 weeks of deployment (Fig. 2). Mean day-length in Papua New Guinea over the trial period was 12 hours 5 mins. The mean voltage of PTT Q was significantly higher (*F* = 21.3, *df*  = 47, *P* = <0.001) in (southern hemisphere) spring (September to November 2006, 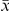 = 4.16 V, *SD*  = 0.04, n = 13) than autumn (March to May 2007, 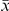 = 3.96 V, *SD*  = 0.04, n = 14) but not summer (December 2006 to February 2007, 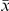 = 4.04 V, *SD*  = 0.07, n = 13) or winter (June to August 2006, 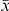 = 4.02 V, *SD*  = 0.11, n = 8), (Fig. 2).

### Study 3: Location Class Error

The mean error between Argos reported locations and actual PTT locations was 199 m (*SD*  = 128, n = 43) for LC error 3, 306 m (*SD*  = 165, n = 17) for LC error 2, 1403 m (*SD*  = 2346, n = 7) for LC error 1, and 3679 m (*SD*  = 1523, n = 2) for LC error class 0.

### Study 4: Captive bat studies

In the first trial, none of the five *P. alecto* showed any serious adverse effect of collaring. All were observed to eat, groom, move, and otherwise behave normally. Bats 1–3 showed mild reddening of the skin underneath the collar until day 9, but not subsequently. Bat 4 lost one rivet from its collar on day 7. Bat 5 developed a 7 mm diameter callus either side of the trachea from day 9. All bats had re-orientated their 180° rotated collars on day 10.

In the second trial, of the 8 *P. scapulatus* and 2 *P. conspicillatus*, one *P. conspicillatus* continually licked at the collar and his neck in the 48 hrs post-collaring. The collar was removed and the bat took no further part in the trial. All nine other bats appeared to tolerate the collars well, and at six weeks post-collaring, no negative impacts were evident. This period coincided with the breeding season of *P. scapulatus*, and mating was observed to occur normally and unencumbered for both collared males and females. At 8 weeks post-collaring, two *P. scapulatus* were found to have moderate moist dermatitis and ulceration on the ventro-lateral aspect of the neck. Both collars were removed and both bats recovered fully with treatment. At 14 weeks, the collar of the remaining *P. conspicillatus* was removed prior to its release to the wild. Mild hair loss was evident. At 15 weeks, two collars (without bats) were found caught on a metal hook which suspended feeding stations from the roof of the enclosure. The respective bats were unharmed and no collar-related lesions were evident. At 18 weeks, three of the remaining four bats had mild hair loss under the collar, and the fourth had severe hair loss and minor skin abrasion; the collar was removed from the latter. At the end of the trial, 28-weeks post collaring, the collars were removed from the remaining three bats: two had the mild hair loss previously evident; the third had a severe and extensive suppurative dermatitis on the ventro-lateral aspects of the neck, and had lost 25% of bodyweight. It recovered fully with treatment.

## Discussion

### Study 1: Collar Design

This study incorporates the largest sample size (n = 20) of satellite transmitters deployed on bats to date. However, due to the high cost of PTTs, (∼$3000 USD), the number of replicates was limited, which reduced our ability to control variables such as PPT type and species.

Premature cessation of transmission (defined by us as <100 days) occurred with 10/20 (50%) deployments, and with all collar designs, with both battery and solar PTTs, and with all species (albeit that limited replicates preclude statistical analysis). This outcome is a major constraint to data collection and warrants discussion. Premature cessation of transmission could be due to a number of factors. The possibility that 50% of transmitters could be technically faulty is both disconcerting and improbable, although it is tempting to attribute the simultaneous failure of PTTs O and P (with identical deployment histories) at 47 days to battery/charge issues. Transmitter loss is another possible factor, however it is again improbable that 50% of collars would either lose their PTT or be lost from the bats with PTT attached. The captive bat trials suggest that collars can be associated with negative health impacts and loss of body condition (albeit in a minority of individuals), so premature bat mortality cannot be discounted as a possible explanation for early cessation of transmissions. Such premature mortality might plausibly result from secondary infection of lesions such as those seen in our captive trials, or from increased vulnerability to hunting. Note that Bat H was trapped and killed by a hunter in Peninsular Malaysia 42 days after release. Regardless of the cause, premature cessation of transmission is a key challenge to telemetry [33]. The PTTs we used were equipped with an activity sensor, however the sensor does not distinguish between a flying fox that has died, a collar that has fallen off, or a PTT that has developed a technical fault. Some of our PTTs were equipped with a ‘ground-track’ capability, wherein the PTT emits a VHF signal upon ‘mortality’, theoretically allowing the use of a handheld antenna and receiver to retrieve the PTT. However, in many cases, retrieval is likely to be impractical due to remoteness or inaccessibility.

A fundamental tenet of telemetry studies is that instrumentation should not interfere with normal behavior; the consequence of such interference is potentially more significant in volant species than in terrestrial species [34]. It is our contention that flying foxes across their range are under increasing ecological pressure, and that energetic margins are slim. Indeed we sought to deploy transmitters only on mature male bats (although bat S was a mature female), mindful of the additional seasonal burden that breeding females are subject to in carrying young pups. Thus, the collar and bib design (Design 1), accommodating large PTTs, cannot be recommended. In Designs 2 and 3, modification of PTTs to ensure that the antenna lay parallel to the axis of the spine allowed us to achieve the optimum plane for signal transmission by allowing the animals body to act as a ‘ground’. However in Design 2, the sheepskin lining became matted over time when contaminated with secretions or wastes, reducing its effectiveness to offer protection from the leather, and potentially providing a nidus for bacterial or fungal skin infection. Also, the curved aluminum mounting bracket used in Design 2 could increase the likelihood of animals becoming snagged and suffering injury and/or collar loss**.** Filling **t**he gap between the top and bottom faces of the bracket would remove the increased risk, but would also add to overall weight. In our experience, the need for a bracket is dependent on two factors: the circumference of the bat's neck, and the length of the (horizontally aligned) PTT. If affixing the PTT directly to the collar ‘deforms’ the circular shape of the collar relative to the bat's neck, a bracket should be considered. We suggest that this is likely when the length of the PTT approached the diameter of the bat's neck. Our observations suggest that the tendency for collar flexing when a bracket is incorporated in the design is independent of PTT model, and is fundamentally due to the centre of gravity of the transmitter being placed out from the bat's neck, albeit that a PTT with a high (vs low) profile would exacerbate this. A wider collar may reduce this flex, but may also increase the likelihood of rub-induced trauma to the angle of the jaw and the neck, and is not recommended.

In Design 3, the compact ‘implantable’ PTT attached directly to the collar makes the bracket redundant, and the non-absorbent neoprene lining protects from abrasion and infection. We believe this design best addresses the key issues of weight, size, profile and comfort.

In this study, we anaesthetized all bats prior to fitting collars, based on our previous experience in handling *Pteropus* species. The reasons are three-fold: firstly, to ensure an optimal collar fit - under anaesthetic, the animal is in a relaxed state and the natural proportions of the neck can be ascertained; secondly, from a workplace safety perspective – anaesthesia removes the risk of bite incidents while working close to the mouth; thirdly, from an animal welfare perspective - fitting the collar correctly takes time and manipulation of the head and neck of the bat, and anaesthesia avoids any associated stress.

We have not investigated built-in collar release/failure mechanisms in this study. There are sound welfare and ethical bases for this consideration, but significant practical challenges exist with some species in achieving a tightly pre-determined time of release. We secured collars with two rivets in an endeavor to ensure that premature collar loss did not occur, and relied on the weathering and deterioration of the collar leather to facilitate eventual collar failure and collar/transmitter shedding. The leather collars we used were typically 11 mm wide and 1.4 mm thick; with two hole punches of 3–4 mm approximately 15 mm apart to accommodate the rivets, the remaining 3–4 mm of leather on either side of the hole being the likely point of failure over a one-two year timeframe, or if a bat were caught in vegetation by the collar. Interestingly, our captive trials demonstrated both premature rivet loss (in one of five bats after 7 days, the rivet having been incorrectly fastened) and the ability for bats to slip out of the collar when it was caught on a snag (two of ten bats, seven weeks post-collaring). However, individual leather and environmental variables make precise quantification of collar failure unfeasible.

### Study 2: Solar-powered PTT assessment

Our original aim was to obtain a multi-year transmission period to examine long-term movement of flying foxes. Our initial battery-powered PTTs offered a maximum one-year operational life even with modest duty cycles. Solar powered PTTs (35 g) have been used successfully to collect data on bird movement for 3 years [35]. Our pre-deployment trial indicated that the 12 g solar powered PTTs had the ability to charge the battery when exposed to both direct and indirect sunlight. This is a fundamental issue given that flying foxes spend the daylight hours hanging in an inverted vertical position in trees, and indicated that solar-powered PTTs could theoretically be used in flying foxes. However, in practice, the efficiency of the solar panel charging, and transmitter activity varied among species. Solar powered PTTs were deployed on three species (*P. alecto, P. vampyrus and P. scapulatus*), but only those on *P. alecto* were able to maintain a voltage sufficient for operation. This species typically roosts on the upper and outer branches, and at low to moderate colony densities, where the solar panels are plausibly exposed to extended periods of direct and indirect sunlight; in contrast, *P. scapulatus* tend to roost low to the ground, beneath dense foliage, and in large and dense colonies, such that panels are likely exposed to infrequent and indirect sunlight. Thus, when *P. scapulatus* is included in our analysis, the median period of transmitter activity for solar-powered PTTs is 68 days; when *P. scapulatus* is excluded, the median period is 225 days. As previously noted, a hunter killed the single *P. vampyrus* fitted with a solar powered PTT 42 days after deployment.

The counterintuitive finding of PTT Q having a higher mean voltage in spring than summer, despite an hour less sunlight, could plausibly be explained by the heavy and frequent monsoonal rains on the southern coast of New Guinea during this period. This would reduce both direct and indirect sunlight, and also drive flying foxes to seek shelter in denser vegetation. Thus, in addition to roosting habitat and behavior, the nature and pattern of prevailing weather should also be considered before employing solar-powered PTTs.

### Study 3: Location Class Error Trial

We found the range of actual errors to be similar to, but slightly higher than, the LC errors stated by Argos. These findings corroborate those of White & Sjoberg [25], who, using satellite relay dataloggers and differentially corrected GPS units, also found the accuracy of location class estimates to be similar to those reported by Argos.

### Study 4: Captive bat studies

Our first trial aimed to identify any adverse impacts of collaring, using Design 2 collars mounted with mock 12 g solar powered PTTs. The transient reddening of the skin under the collars on Bats 1–3 (lined and unlined ‘chrome’ leather) is likely due to minor irritation prior to bats becoming accustomed to their new collars. The callused skin over the throat of Bat 5 suggests more chronic irritation, likely due to the less pliable nature of the ‘veggie-tan’ leather collar, which should thus be considered unsuitable for collars. In our second, long-term captive trial, we found that the sheepskin lining used in these bats collars became matted, lost resilience over time and caused moderate to severe ventral and lateral neck ulceration in 3/10 bats. These lesions were evidently sufficient to affect behavior and are a clear ethical and animal welfare concern. That all three bats affected were *P. scapulatus* suggests a species-level effect. A plausible explanation is that the smaller size of *P. scapulatus* means that a collar width appropriate for a larger (and longer necked) species might cause constant abrasion to the angle of the jaw of a smaller (and shorter necked) species. The involvement of the angle of the jaw evident in Fig. 1b supports this explanation. Thus collar width may be another important consideration in collar design.

The observation on Day 10 of the first captive trial (after the intentional rotation of the mock PTTs on Day 9) that all five flying foxes had re-orientated the PTT to the dorsal aspect, established that flying foxes were able to reposition the PTT, presumably for comfort. Fortunately, this apparent bat-preferred orientation is also optimal for transmission and for maximizing solar panels exposure to sunlight. Observations in the second trial support those of the first: all bats had dorsal alignment of the PTT throughout the 28 week trial.

### Conclusion

Satellite telemetry is undoubtedly a valuable tool for studying the long-distance movement of bats. However, it is evident that both data validity and maximum transmission period are dependent on optimal PTT/collar configuration. Our study suggests that no one design configuration suits all scenarios, and that (in addition to the study objectives) both biological variables (species size/weight, roosting habitat and behavior) and climatic variables (day-length, rainfall pattern) are key considerations in transmitter and collar configuration. However, a number of specific insights on optimizing PTT/collar configuration flow from our work: a neoprene collar, a compact PTT mounted directly to the collar, and an aerial orientation perpendicular to the PTT along the dorsum. In addition, we support the recommendations of others that the combined collar/PTT weight not exceed 5% of bat body weight. This is most practically achieved by restricting collaring to adult male bats.

For battery-powered PTTs, it is evident that, for any given bat size/weight and PTT dimension/weight, compromise is required between frequency/duration of transmission and the length of the study. Future reduction of PTT size and weight (largely battery-dependent) may alleviate these and other design issues.

Finally, it is evident that there is considerable variability among species, and that the potential exists for significant ethical and animal welfare issues.
